# High CD56^++^CD16^-^ natural killer (NK) cells among suboptimal immune responders after four years of suppressive antiretroviral therapy in an African adult HIV treatment cohort

**DOI:** 10.1186/1471-2172-15-2

**Published:** 2014-01-31

**Authors:** Lois Bayigga, Rose Nabatanzi, Prossy Naluyima Sekiziyivu, Harriet Mayanja-Kizza, Moses R Kamya, Andrew Kambugu, Joseph Olobo, Agnes Kiragga, Sam Kirimunda, Moses Joloba, Damalie Nakanjako

**Affiliations:** 1 Department of Medical Microbiology, Makerere University College of Health Sciences, Kampala, Uganda; 2 Makerere University Walter Reed Project, Makerere University College of Health Sciences, Kampala, Uganda; 3 Department of Internal Medicine, Makerere University College of Health Sciences, Makerere University, Kampala, Uganda; 4 Infectious Diseases Institute, Makerere University, Kampala, Uganda

**Keywords:** Natural killer cells, Suppressive antiretroviral therapy, HAART, Suboptimal immune recovery, HAART, Sub-saharan Africa

## Abstract

**Background:**

Up to 40% of HIV-infected individuals receiving Highly Active Antiretroviral Therapy (HAART) have poor CD4+ T-cell recovery. The role of natural killer (NK) cells in immune recovery during HAART is not well understood. We described the profiles of NK cell subsets and their expression of activating receptor, NKG2D and cytotoxicity receptor NKp46 among suboptimal immune responders to despite four years of suppressive HAART.

**Methods:**

A case control study utilized frozen peripheral blood mononuclear cells (PBMC) from a cohort of HIV-infected adults that initiated HAART in 2004/5, at CD4 < 200 cells/μl. Cases were ‘suboptimal’ responders; patients within the lowest quartile of CD4+ T-cell reconstitution, with a median CD4 count increase of 129 (-43-199) cells/μl (difference between CD4 count at baseline and after 4 years of HAART) and controls were ‘super-optimal’ responders; patients within the highest quartile of CD4 T-cell reconstitution with a median CD4 count increase of 528 (416-878) cells/μl). Expression of NK cell lineage markers (CD56^+/-^CD16^+/-^) and receptors NKG2D and NKp46, was measured among PBMC from 29 cases of ‘suboptimal’ responders’ and 23 controls of ‘super-optimal responders’, and compared among ‘suboptimal’ and ‘super-optimal’ responders. NK cell populations were compared using the Holm Sidak multiple comparison test and p values < 0.05 were considered statistically significant. Data was analyzed using FLOWJO and GraphPad Prism 6.

**Results:**

‘Suboptimal responders’ had a higher proportion of cytokine producing CD56^++^CD16^+/-^ (CD56^bri^) NK cells than the ‘super-optimal responders’ *p = 0.017*, and CD56^neg^ NK cells were lower among suboptimal than super-optimal responders (*p = 0.007).* The largest NK cell subset, CD56^dim^, was comparable among suboptimal responders and ‘super-optimal immune responders’. Expression of NKG2D and NKp46 receptors on NK cell subsets (CD56^bri^, CD56^neg^ and CD56^dim^), was comparable among ‘suboptimal’ and ‘super-optimal’ immune responders.

**Conclusions:**

The pro-inflammatory CD56^++^CD16^--^ NK cells were higher among ‘suboptimal’ responders relative to ‘super-optimal’ responders, despite four years of suppressive HAART. Alteration of NK cell populations could inhibit host immune responses to infections among suboptimal responders. We recommend further analysis of NK cell function among suboptimal immune responders in order to inform targeted interventions to optimize immune recovery among HAART-treated adults.

## Background

Suboptimal immune recovery occurs in up to 40% of HIV-infected individuals receiving long-term Highly Active Antiretroviral Therapy (HAART) in sub-Saharan Africa (SSA) [1]– [3]. The exact mechanisms for suboptimal immune recovery are not fully established, although the phenomenon has been associated with low nadir CD4 count at HAART initiation, irreversible fibrosis of the reticulo-endothelial system during advanced HIV disease, persistent T-cell activation and immune exhaustion, among other factors [2,4,5]. There is limited data on how HIV-associated dysfunction of the innate immune system influences immune recovery, in particular Natural Killer (NK) cells that are known to participate in the initiation and development of adaptive immune responses. NK cells also participate in host innate responses to viral and intra-cytoplasmic bacterial infections [6-8], and may have a role in immune recovery among HAART-treated HIV-infected adults. HIV-associated NK cell dysfunction has been reported in association with severity of HIV disease [9] and the impaired immune responses associated with HIV/AIDS [10,11]. In addition, increased NK cell activation and degranulation have been associated with Immune Reconstitution Inflammatory Syndrome (IRIS) and TB/HIV co-infections [12,13], which contribute to HIV-associated morbidity and mortality during HAART [14]– [16]. There is a need to understand the role of innate immune dysfunction in post-HAART immune recovery, to inform therapeutic advances to optimize HIV treatment outcomes. This paper explores the association of NK cells with immune recovery during suppressive HAART in an African HIV treatment cohort.

The role of the innate immune system in HIV immune-pathogenesis has been explored with particular focus on NK cell subsets, function and expression of receptors [11,17]– [19]. Three distinct subsets of NK cells are recognized in human peripheral blood; CD56^bri^, CD56^dim^ and CD56^neg^; categorized according to the expression of NK cell lineage markers CD56 and CD16 [20,21]. CD56^bri^ are pre-dominantly cytokine producing cells and CD56^dim^ are mainly cytotoxic [22]. NK cell function is directed by a complex repertoire of activating and inhibitory natural cytotoxicity receptors (NCRs), such as NKp46, NKp30 and NKp44, as well as NKG2D, CD16, 2B4 and NKp80 [22]. During HIV infection, NK cells are directly infected [23] and the distribution of NK cell subsets is altered [20]; with an expansion of CD56^neg^ among viremic patients [20]. In addition, HIV causes up-regulation of inhibitory natural killer receptors (iNKRs) leading to impairment of NK cell lysis of virally-infected cells [11]. Antiretroviral therapy reverses the effects of HIV infection on NK cells; however, there is no consensus on the degree to which suppression of HIV replication restores NK cell function [10]. We hypothesized that the distribution and function of NK cell subsets differs among individuals with poor versus excellent CD4+ T-cell recovery during antiretroviral therapy. This study describes the profiles of NK cell subsets and their expression of activating receptors, NKG2D and cytotoxicity receptor NKp46, among individuals with poor CD4 T-cell reconstitution relative to individuals with excellent CD4 T-cell count reconstitution after four years of suppressive HAART. Our results highlight the need for studies to further understand the short and long-term recovery of the innate immune system including NK cell function among African HAART-treated HIV-infected patients.

## Methods

### Study design and participants

**Figure 1:**
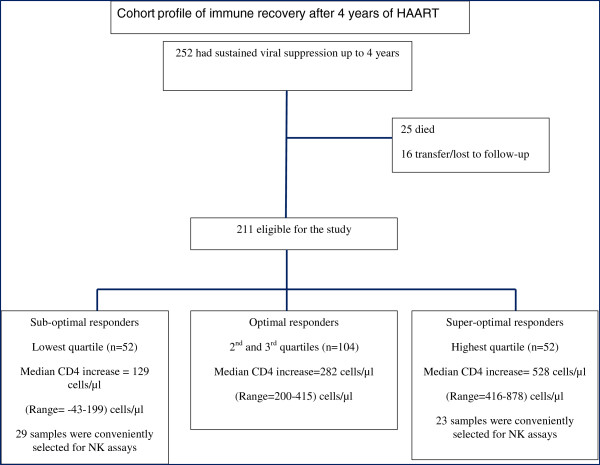
**Profile of patients on antiretroviral therapy in the Infectious Diseases research cohort.** Natural Killer (NK) cell populations and expression of activating receptors were compared among ‘suboptimal’ immune responders (cases) and ‘super-optimal’ immune responders (controls).

Using a case-control study design, NK cell populations and their receptors were evaluated among ‘suboptimal’ immune responders (cases) and ‘super-optimal’ immune responders (controls) after four years of HAART within the Infectious Diseases Institute (IDI) research cohort. This study utilized frozen peripheral blood mononuclear cells (PBMC) that were collected from a Ugandan Adult HIV Treatment Cohort that was previously described [4]. Between April, 2004 and April, 2005, 559 HAART-naïve HIV-infected adults were consecutively initiated on HAART, and enrolled in a prospective observational research cohort. HIV RNA viral load and CD4 count measurements were done every six months and viral suppression was considered at <400 copies/ml. After four years of HAART, individuals that were still in care, with sustained viral suppression and no history of opportunistic infections in the six months preceding the study, were included in the study. Individual CD4 count increases (difference between CD4 count at HAART initiation and CD4 count after 4 years of HAART) of study participants were grouped into quartiles. ‘Suboptimal’ responders were patients within the lowest quartile of CD4+ T-cell reconstitution with a median CD4 count increase (minimum-maximum) of 129 (43–199) cells/μl and ‘super-optimal’ responders were patients within the highest quartile of CD4 T-cell reconstitution with a median CD4 count increase of 528 (416–878) cells/μl); see Figure 1. All participants in the parent study provided written informed consent for storage of biological samples for further immunological studies. This study was approved by the School of Biomedical Sciences Institutional review board with final approval by the Uganda National Council for Science and Technology. Laboratory assays were conducted at the Immunology laboratory Department of Microbiology, College of Health Sciences and the IDI translational laboratory that is within the same complex. Flow cytometry assays were conducted using an eight-colour FACS Canto II (BD Biosciences) Flow cytometer.

### Cell surface staining

**Figure 2:**
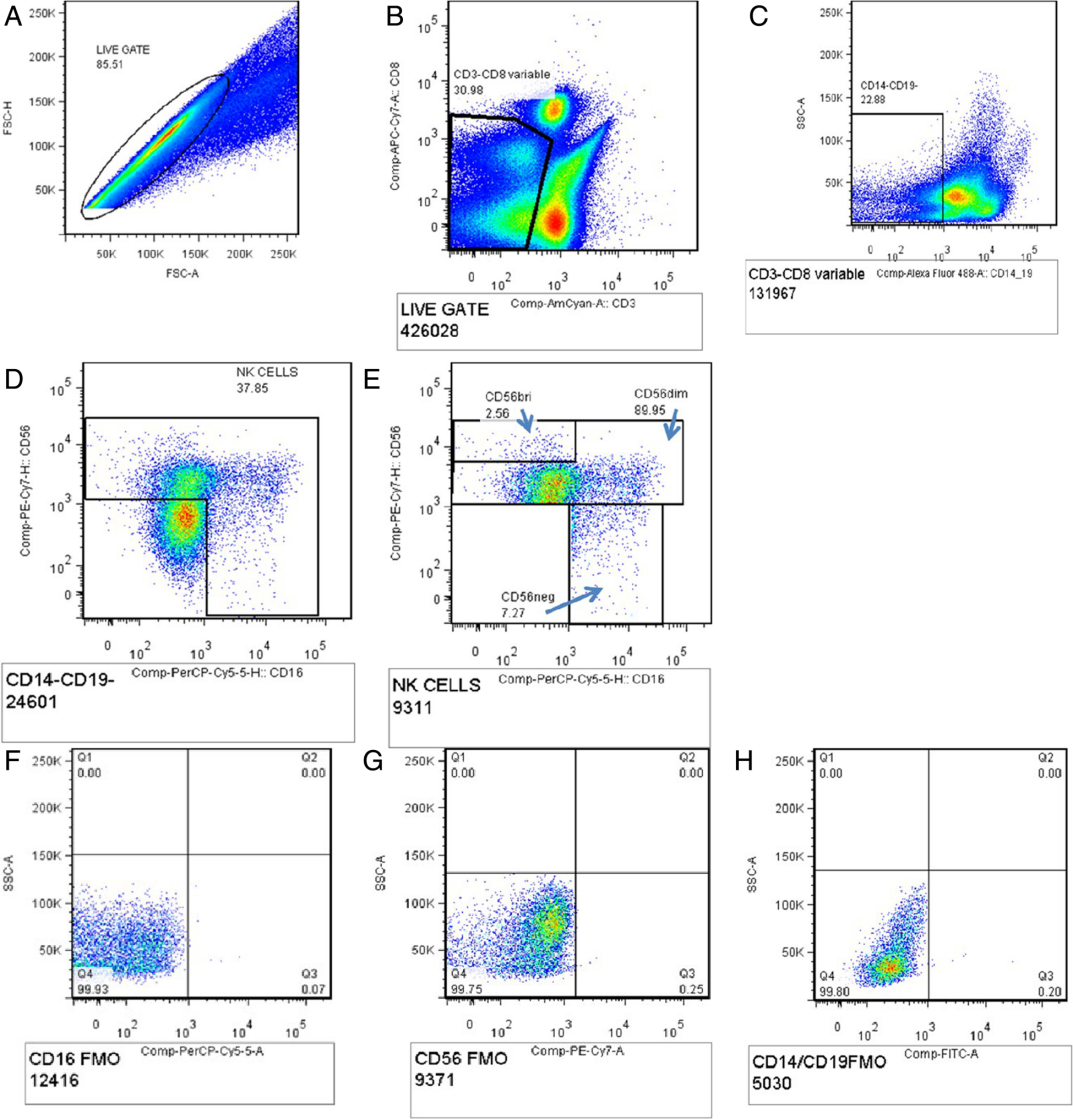
**Gating strategy for NK cells. A**- Total lymphocyte population, **B**- CD3 negative CD8 variable NK cells gated from the total lymphocyte population, **C**- CD14-/19- cells, **D**- total NK cell population gated off the CD14-/19- cells, **E**- different NK cell subsets; CD56^bri^ (cytokine-producing NK cells)-CD56^++^ CD16^-^, CD56 ^dim^ (cytotoxic NK cells) -CD56^+^CD16^+/-^, CD56 ^neg^ (cytotoxic NK cells)-CD56^-^CD16^+^, **F**- CD16 PerCP-cy5.5 FMO, **G**- CD56 PE-Cy7 FMO and **H**- CD14/19-FITC FMO.

**Figure 3:**
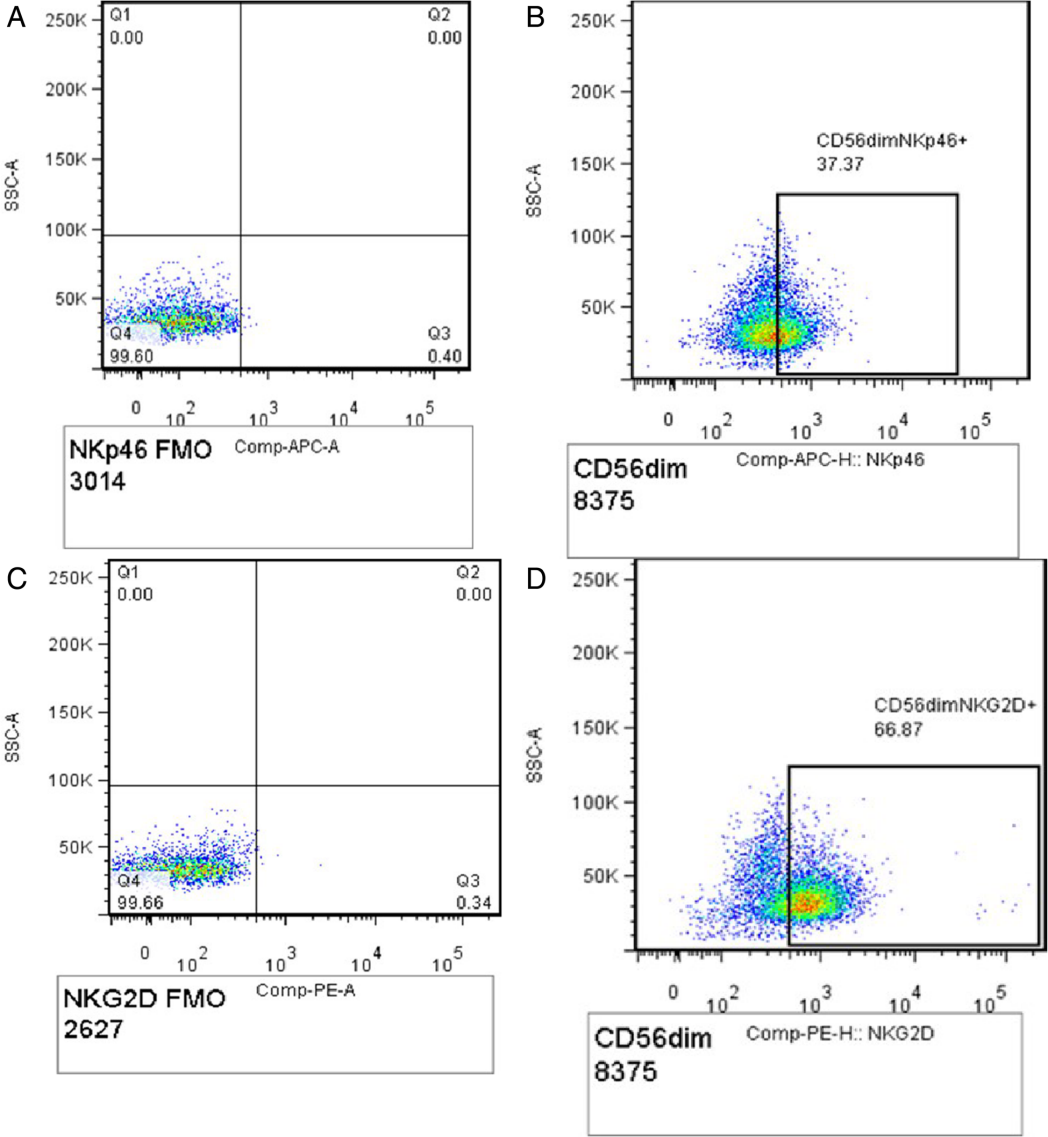
**Gating strategy for expression of NKp46 and NKG2D by CD56 +** ^**dim **^**NK cells: Panel A shows NKp46 APC FMO, B shows CD56**^**dim **^**expressing NKG2D (CD56**^**+**^**CD16**^**+/-**^**NKG2D**^**+**^**); C shows NKG2D PE FMO and D shows CD56**^**dim **^**expressing NKG2D (CD56**^**+**^**CD16**^**+/- **^**NKG2D**^**+**^**).**

This study utilized frozen PBMC that were previously collected using the Ficoll-Paque method and stored in liquid nitrogen [24]. PBMC were thawed in a water-bath at 37°C for one minute and thereafter, washed and re-suspended in RPMI media containing 10% v/v fetal calf serum before surface staining. PBMC viability was evaluated using trypan blue dye and viable cells were counted under microscopy. Minimum cell viability was 80%. Cells were rested for four hours, and subsequently stained with the following anti-human monoclonal antibodies; CD14/19 FITC CD3 Amcyan, CD8 APC-Cy7, CD16 PerCP Cy5.5, CD56 PE Cy7, NKG2D PE and NKp46 APC (BD Biosciences, San Jose CA). At least 50,000 events in the CD3-negative gate were collected. Gating was standardized and set using fluorescence minus one control (FMOs) for CD14/19, CD16, CD56, NKG2D and NKp46. NK cells were identified as CD3^-^negative, CD14^-^/CD19^-^ and NK cell subsets were identified by co-expression of CD56 and CD16 on NK cells; CD56^bri^ (CD56^++^CD16^-^), CD56^INTERMEDIATE^ (CD56^+^CD16^-^), CD56^dim^ (CD56^+^CD16^+^) and CD56^neg^ (CD56^-^CD16^+^); see Figure 2. Expression of NK receptors NKG2D and NKp46 was defined by the percentage of NKG2D + and NKp46+ NK cell subsets (Figure 3).

### Statistical analysis

Data obtained from flow cytometry was analyzed using FLOWJO version 7.6.3 software (TreeStar, San Carlos, CA), exported to Excel spreadsheets and subsequently analyzed using GraphPad Prism 6. NK cell populations and expression of NK surface receptors NKG2D and NKp46 were compared among ‘suboptimal’ and ‘super-optimal’ immune responders, using the Holm Sidak multiple comparison test and p values < 0.05 were considered statistically significant.

## Results

### NK cell subsets in peripheral blood after four years of suppressive HAART

**Figure 4:**
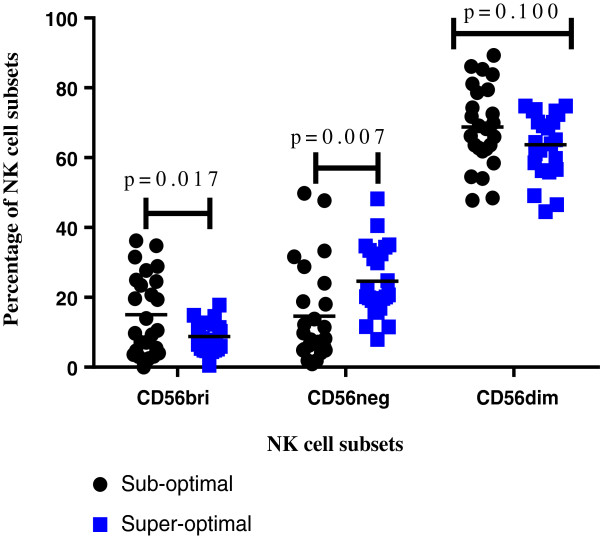
**Comparison of NK cell subsets among suboptimal and super-optimal immune responders after four years of suppressive antiretroviral therapy.** Figure 4 shows comparison of mean percentages of NK cell subsets (CD56^bri^, CD56^neg^ and CD56^dim^) among sub-optimal and super-optimal responders using the Holm Sidak multiple comparison test.

Overall, the largest NK cell subset was the CD56^dim^; accounting for an average of 59% of NK cells among suboptimal responders and 57% of NK cells among ‘super-optimal responders’. The proportions of CD56^bri^ were the lowest among both sub-optimal and optimal responders (Figure 4). CD56^bri^ (cytokine producing) NK cells were higher in suboptimal than super-optimal responders, *p = 0.017*, and CD56^neg^ NK cells were lower among suboptimal than super-optimal responders (*p = 0.007).*

### Expression of NKG2D and NKp46 receptors on NK cells among ‘sub-optimal’ and ‘super-optimal’ responders

**Figure 5:**
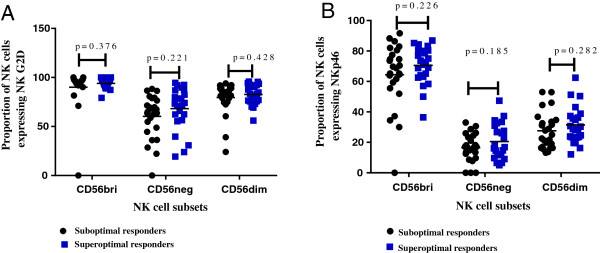
**Expression of NK activating receptors NKG2D and NKp46 on NK subsets among suboptimal and super-optimal responders after four years of suppressive antiretroviral therapy. A** shows NKG2D expression and **B** shows NKp46 expression.

Expression of NKG2D receptor was comparable among suboptimal and super-optimal responders; CD56^bri^ (CD56^++^CD16^-^NKG2D^+^), p = 0.376; CD56^neg^ (CD56^-^CD16^+^NKG2D^+^), p = .0.221 and CD56^dim^ (CD56^+^CD16^+/-^NKG2D^+^), p = 0.428 (Figure 5). Similarly, NKp46 expression was comparable among suboptimal and super-optimal responders; CD56^bri^ (CD56^++^CD16^-^NKp46^+^), p = 0.226, CD56^neg^ (CD56^-^CD16^+^NKp46^+^), p = 0.185 and CD56^dim^ (CD56^+^CD16^+/-^NKp46^+^); p = 0.282 (Figure 5).

## Discussion

This study compared proportions of NK cell subsets as well as expression of activating receptor NKG2D and cytotoxicity receptor NKp46 among adults with ‘suboptimal’ and ‘super-optimal’ immune recovery despite four years of suppressive HAART. We found that CD56^dim^ was the largest NK cell subset among HAART-treated adults, irrespective of immune recovery status. Our data is consistent with previous reports that CD56^dim^ is the largest population of NK cells in peripheral blood and the main cytotoxic NK cells participating in Antibody-dependent cell-mediated cytotoxicity (ADCC); followed by CD56^neg^ and CD56^bri^ NK cells. Whereas the functionally defective CD56-CD16+ (CD56^neg^) population of NK cells expands in viremic versus aviremic patients [11] and is associated with poor cytotoxic function [20], this subset of NK cells was lower among suboptimal responders relative to super-optimal responders. Given that our study participants had received suppressive HAART for four years, our data suggests that HIV-associated expansion of the dysfunctional CD56^neg^ population was no longer significant. These results mirror previous reports that initiating HAART during acute HIV infection prevented further decline in NK cell subsets and improved NK cell function [25]. It is therefore likely that initiating HAART earlier in HIV disease when the immune systems are still robust, as recommended in the 2013 WHO guidelines [26], may result into faster recovery of HIV-associated NK cell dysfunction; among other benefits.

The CD56^++^CD16^-^ (CD56^bri^) subset, functionally cytokine producers, was higher among ‘suboptimal’ responders relative to ‘super-optimal’ responders. The high numbers of cytokine producing NK cells among ‘suboptimal’ responders may be reflective of the persistently high levels of immune activation that were previously documented among suboptimal responders in our cohort [4]. Immune activation has been associated with high production of inflammatory cytokines and increased turn-over of T-cells, B lymphocytes, NK cells and accessory cells [27,28]. Immune activation in the first few years of HAART-mediated viral suppression predicted long-term CD4+ T-cell recovery after 15 years of antiretroviral therapy. It is likely that pre-HAART immune activation, not only predicts mortality during HAART [29], but also predicts suboptimal immune recovery including suboptimal reversal of the HIV-associated NK cell dysfunction. We therefore postulate that controlling immune activation among HIV-infected individuals may also stabilize the NK cytokine producing cells, modulate their immune function and subsequently optimize short and long-term immune recovery during antiretroviral therapy.

Expression of NKG2D and NKp46 receptors by NK cells was comparable among suboptimal and super-optimal responders. NK activating receptors correlate with the NK effector function [30,31], so it is likely that NK effector function is comparable among suboptimal and super-optimal responders. We could attribute this result to suppressive HAART that has provided partial immune recovery during the first four years. It has been previously shown that HAART modulates NKG2D receptor expression among HIV-infected viremic individuals [10,13,21]. Increased NKp46 expression on NK cells was previously shown to correlate with HIV-1 disease severity among HIV-infected children [9], although its role in immune recovery is not yet well understood. Although we did not perform NK functional assays, previous data shows that abnormal expression of NK activating and inhibitory receptors was associated with impaired cytolytic function [11]. In addition, reduced surface expression of the NK cytotoxicity receptor, NKp46 was associated with poor cytolytic function during viremic HIV disease [21]. Given that NKG2D and NKp46 expression was similar in the ‘suboptimal’ and ‘super-optimal’ immune responders, it is likely that these receptors are not involved in the mechanisms that lead to poor CD4+ T-cell reconstitution in HIV-infected adults receiving HAART unless functional assays reveal significant differences. In addition, NK cell activation is controlled by a dynamic balance between complementary and antagonistic pathways [31,32]. We did not evaluate NK cell surface inhibitory receptors that antagonize activating pathways through protein tyrosine phosphatases (PTPs), therefore our data is not conclusive on the NK cell activation status in the study population.

### Implications of the study

Our results imply that after four years of suppressive antiretroviral therapy, the HIV-associated NK cell dysfunction was only partially restored, with a predominant CD56^dim^ (CD56 + CD16-/+) population and a high CD56^bri^ (CD56^++^CD16^-^) NK cell population among suboptimal responders. The high CD56^bri^, functionally cytokine producers, among suboptimal responders may be reflective of the persistently high levels of immune activation previously described in the same cohort [4]. We postulate that earlier initiation of HAART and control of immune activation could contribute to faster and more comprehensive recovery of the immune system. It is also important to note the trend shown that specific T-cell subsets recover faster, while other subsets require longer periods of suppressive HAART. Given the significant cytokine producing function of CD56^bri^ NK cells, it might be worthwhile to further investigate NK cell dysfunction among individuals that initiate HAART at CD4 < 500 cells before severe damage of the immune system, as well as potential interventions to enhance comprehensive immune recovery. In addition, increased NK cell degranulation capacity was significantly associated with Immune Reconstitution Inflammatory Syndrome (IRIS) among HIV/TB co-infected individuals in Cambodia, with activating receptor expression higher among IRIS patients relative to non-IRIS patients [17]. Similarly NK cell activation was shown to distinguish Mycobacterium tuberculosis-mediated IRIS from chronic HIV and HIV/TB co-infection [12]. Thus, further examination of the mechanisms of NK cell dysfunction, co-infections and suboptimal recovery is critical for suboptimal immune responders that remain at risk of life-threatening opportunistic infections [1,24].

### Limitations

We did not perform NK cell function assays due to logistical limitations. In addition, this study was limited to the extremes of immune recovery (suboptimal and super-optimal immune responders), and did not include average responders. Our results, however, highlight the need for NK function assays to conclusively ascertain defects in NK cell effector function that might be relevant to immune responses to viral and bacterial infections among HAART-treated HIV-infected adults.

## Conclusion

The pro-inflammatory CD56^++^CD16^-^ NK cells were higher among ‘suboptimal’ responders relative to ‘super-optimal’ responders, despite four years of suppressive HAART. Alteration of NK cell populations could inhibit host immune responses to infections among suboptimal responders. We recommend further analysis of NK cell function among suboptimal immune responders to inform targeted interventions to optimize immune recovery among HAART-treated adults.

## Competing interests

The authors declare that they have no competing interests.

## Authors’ contribution

LB, DN, HMK and JO made substantial contribution to the conception, design and interpretation of the data. LB, DN, MJ, RN, SK and PNS made substantial contribution to the data collection, flow cytometry assays and data analysis. DN, MRK, AK and AK contributed substantially to the clinical cohort from which the samples were collected. LB and DN drafted the manuscript. All authors reviewed the manuscript and approved the final version for publication.
